# Murine Anti-vaccinia Virus D8 Antibodies Target Different Epitopes and Differ in Their Ability to Block D8 Binding to CS-E

**DOI:** 10.1371/journal.ppat.1004495

**Published:** 2014-12-04

**Authors:** Michael H. Matho, Natalia de Val, Gregory M. Miller, Joshua Brown, Andrew Schlossman, Xiangzhi Meng, Shane Crotty, Bjoern Peters, Yan Xiang, Linda C. Hsieh-Wilson, Andrew B. Ward, Dirk M. Zajonc

**Affiliations:** 1 Division of Cell Biology, La Jolla Institute for Allergy and Imunology (LIAI), La Jolla, California, United States of America; 2 Department of Integrative Structural and Computational Biology, The Scripps Research Institute, La Jolla, California, United States of America; 3 Howard Hughes Medical Institute, Division of Chemistry and Chemical Engineering, California Institute of Technology, Pasadena, California, United States of America; 4 Department of Microbiology and Immunology, University of Texas Health Science Center, San Antonio, Texas, United States of America; 5 Division of Vaccine Discovery, La Jolla Institute for Allergy and Immunology (LIAI), La Jolla, California, United States of America; Washington University, United States of America

## Abstract

The IMV envelope protein D8 is an adhesion molecule and a major immunodominant antigen of vaccinia virus (VACV). Here we identified the optimal D8 ligand to be chondroitin sulfate E (CS-E). CS-E is characterized by a disaccharide moiety with two sulfated hydroxyl groups at positions 4′ and 6′ of GalNAc. To study the role of antibodies in preventing D8 adhesion to CS-E, we have used a panel of murine monoclonal antibodies, and tested their ability to compete with CS-E for D8 binding. Among four antibody specificity groups, MAbs of one group (group IV) fully abrogated CS-E binding, while MAbs of a second group (group III) displayed widely varying levels of CS-E blocking. Using EM, we identified the binding site for each antibody specificity group on D8. Recombinant D8 forms a hexameric arrangement, mediated by self-association of a small C-terminal domain of D8. We propose a model in which D8 oligomerization on the IMV would allow VACV to adhere to heterogeneous population of CS, including CS-C and potentially CS-A, while overall increasing binding efficiency to CS-E.

## Author Summary

Vaccinia virus (VACV) is an orthopox virus and considered the gold standard of vaccines as it was used to eradicate smallpox from the human population. Inoculation with VACV leads to a strong B cell immune response and the production of potent antibodies that simultaneously target several envelope proteins of the virus. Among those viral proteins, D8 is an adhesion molecule that binds chondroitin sulfate, a glycosaminoglycan, on the host cell surface. Here, we identified chondroitin sulfate E (CS-E), as the preferred ligand for D8 and assessed the role of a panel of anti-D8 antibodies in preventing D8 binding to CS-E. We further mapped the binding site of each antibody on the D8 surface to reveal the targeted epitopes. Finally, using several truncated D8 constructs, we identified that the C-terminal domain of D8 that is not involved in CS-E binding is in fact involved in oligomerization of native D8 *in vitro* and likely, also on the virion, as a means of increasing binding affinity to increase viral adhesion to CS on the host cell.

## Introduction

Vaccinia virus (VACV) is a low virulence orthopoxvirus that was used to eradicate smallpox [1]. Immunization with VACV leads to the production of potent protective antibodies that target the VACV envelope proteins A27, A33, B5, D8, H3 and L1, among others [1]. VACV has two forms of infectious virions. The intracellular mature virion (IMV) is the most abundant form of VACV and mainly responsible for viral spread between hosts. A27, D8, H3 and L1 are expressed on the outer membrane of IMV. A33 and B5 are embedded in the more fragile extracellular enveloped virion (EEV), which has an additional host cell derived envelope. EEV is thought to be involved in cell-to-cell spread within the host and is critical for virulence. Antibody responses against VACV potently target both infectious forms of the virus, likely contributing to the efficacy of the smallpox vaccine [2]. Among the VACV envelope proteins, A27, H3 and D8 are viral adhesion molecules that bind to glycosaminoglycans (GAG) for attachment to host cells. GAGs are linear polysaccharides with repeating disaccharide units predominantly found on cell surfaces and as constituents of the extracellular matrix [3]. While A27 and H3 interact with heparan sulfate (HS) or heparin (HP) [4], [5], D8 binds to chondroitin sulfate (CS) [6]. Viral adhesion to GAGs represents a major route of entry for a range of pathogens [7]. As a result, GAG adhesion is an early and important step that initiates viral infection.

We have recently determined the crystal structure of the CS adhesion protein D8 [8]. The N-terminal ectodomain contains a carbonic anhydrase fold (CAH, residues 1–234), followed by a smaller domain of unknown function (residues 235–273). A single transmembrane domain (TM, 274–294) and a small intra-virion tail (295–304) constitute the rest of the protein. The CAH domain was suggested to be responsible for CS binding, as it has a central positively charged crevice that complements the negative charge of CS [8].

We have recently identified four different antibody specificity groups among an ensemble of twelve murine monoclonal antibodies specific to D8 [9]. D8 MAbs were first sorted by competitive ELISA according to their targeted epitopes on the basis of which other MAbs they cross-block. Four main specificity groups resulted from this experiment (group I: JE11; II: AB12 and CC7.1; III: BG9.1, BH7.2, EB2.1, EE11, JA11.2, JE10, and JF11; IV: FH4.1 and LA5) [9]. Partial epitope definitions for each of the antibody groups were previously determined. For group I MAbs, the epitope definition was mapped by Deuterium Exchange Mass Spectrometry (DXMS) to residues 10–14 and 80–90 [9]. Peptide ELISA, using 20-mer peptides that overlap by 10 amino acids and cover the entire D8 protein sequence, suggested that group II antibodies target a linear epitope called peptide 58 (residues 91–110), while groups I, III and IV target a conformational epitope [9]. Alanine scanning of peptide 58, and D8 point mutation analysis (PMA) refined the group II epitope to 10 residues (H95, W96, N97, K99, Y101, S102, S103, E106, H110 and D112). While the group III MAb epitope is conformational, group III MAbs can also bind peptide 58, albeit much weaker than group II antibodies. Interestingly, group II and III MAbs cross-block each other. Alanine scanning of peptide 58 gave a partial definition of the group III epitope; it includes, but is not limited to H95, W96, N97, Y101, S103, Y104, E105, E106, and K108. Finally, X-ray crystallography identified the D8 epitope of one MAb of group IV, LA5 [8].

In this study, we used glycosaminoglycan microarrays printed with natural polysaccharides enriched in specific sulfated structures to identify the molecular species of CS that optimally binds to D8 [10], [11]. We further asked whether any of the antibodies that target different binding sites on D8 block D8 adhesion to CS. Lastly, using single particle electron microscopy (EM), we mapped the binding site of a representative antibody of each antibody specificity group on D8. Identification of different binding sites on D8 illuminates the molecular details of the murine antibody response against viral D8. Finally, we discovered two opposing sides of the D8 protein surface that are not targeted by antibodies, likely due to their inaccessibility in the viral membrane, and we propose a model in which D8 forms a hexamer. The hexamerization is mediated by self-association of the previously uncharacterized C-terminal ectodomain (residues 235–273) downstream of the CAH domain.

## Results

### Identification of CS-E as an optimal ligand for D8 and role of MAbs in blocking CS-E adhesion

**Figure 1:**
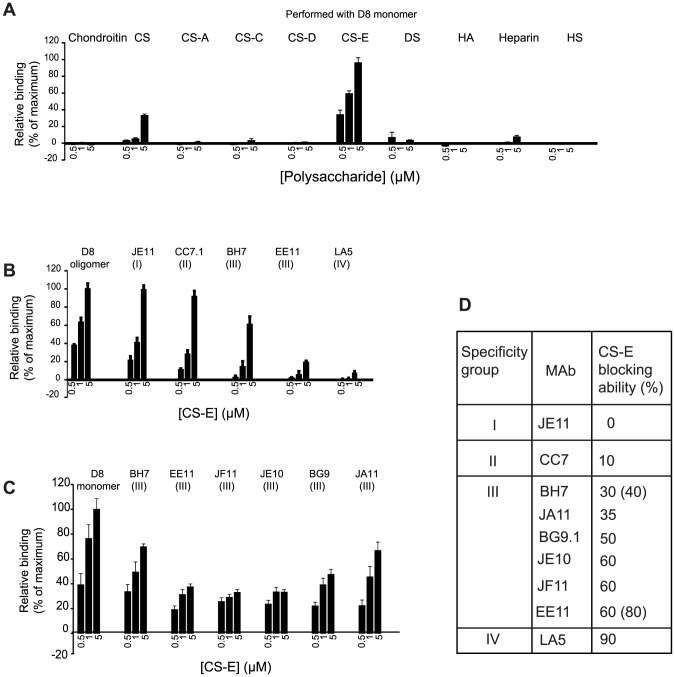
D8 binds to CS-E and anti-D8 MAbs display different levels of competition with CS-E. **A.** GAG microarray performed with monomeric D8 antigen. **B.** MAb/CS-E cross-blocking experiments using representatives of all four antibody specificity groups and oligomeric D8. **C.** MAb/CS-E cross-blocking of group III MAbs **D.** Summary of CS-E cross-blocking abilities of various MAbs. Group III MAbs are characterized by large variations in cross-blocking ability. Microarray binding experiments were performed in triplicate, and the data represent the average of 10 spots per concentration averaged from the three experiments (±SEM, error bars).

To broadly assess D8-GAG interactions, we employed microarrays containing immobilized chondroitin sulfate polysaccharides enriched in specific sulfation motifs (CS-A, -C, -D, and –E), dermatan sulfate (DS), hyaluronic acid (HA), heparin and heparan sulfate (HS). We observed weak binding of monomeric D8 to chondroitin sulfate, a CS preparation containing a mix of sulfation motifs (CS-A, -C, -D, and –E). In contrast, D8 displayed strong concentration-dependent binding to CS-E. Binding to heparan sulfate or chondroitin sulfate with different sulfation patterns was not observed (Fig. 1A).

Having identified the optimal ligand for D8, we then determined the role of anti-D8 antibodies in preventing D8 binding to CS-E using a competition-binding assay. A representative of each of the four antibody specificity groups was first pre-incubated at a saturating concentration with D8 and then tested for binding to CS-E on the GAG microarrays. If the antibody bound at the CS-E binding site on D8, no (or greatly reduced) binding of CS-E would be observed. In contrast, full binding to CS-E would occur for antibodies bound at a site separate from the CS-E binding site. The extent of the CS-E blocking ability (%) of the MAb indicated the degree of overlap of the two binding sites (MAb epitope and CS-E binding site). Cross-blocking power may be explained in terms of intersecting buried surface areas (BSAs), or in terms of competing intermolecular electrostatic interactions.

Group I and II MAb representatives JE11 and CC7.1 did not affect D8 binding to CS-E significantly, indicating that their epitopes do not overlap with the CS-E binding site on D8 (Fig. 1B, D). LA5, a member of group IV, fully abrogates CS-E binding to D8, as we had previously suggested [8] (Fig. 1B, D).

Group II and III MAbs were previously identified to bind peptide 58 (residue 91–110). Residues 91–110 are located on one side of the positively charged crevice of D8 that we previously proposed to be the CS binding site, using computational docking [8]. While group II antibodies did not interfere with CS-E binding, group III antibodies BH7 and EE11 blocked CS-E binding to varying degrees. These results illustrated diversity in this group, which constitutes the majority of the anti-D8 antibodies (eight out of twelve). Despite both group II and III antibodies binding to peptide 58, they surprisingly have different effects on CS-E binding, indicating that they have an overlapping epitope but differ in their binding footprint at the CS-E binding site on D8. Because group II and III MAbs target overlapping epitopes juxtaposing the sugar-binding site, we found such a wide variation in CS-E blocking (BH7 40%, EE11 80%) quite surprising within a single specificity group. As EE11 and BH7 are the most distant representatives of group III antibodies, based on their light chain (LC) sequences (Fig. S1), we next asked whether the other group III antibodies have intermediate CS-E blocking abilities. We tested 6 out of all 8 Group III MAbs. We also used monomeric D8, instead of the oligomer, to avoid potential steric hindrance of antibody binding to D8, which itself could result in CS-E blocking differences (Fig. 1C). Nevertheless, BH7 and EE11 blocked binding to monomeric D8 similarly when compared to oligomeric D8 (30 vs. 40% for BH7 and 60 vs. 80% for EE11). All other analyzed Group III MAbs fall within the same CS-E blocking range (35–60%), indicating subtle differences in their binding footprint at the CS-E binding site of D8 (Fig. 1C, D). Since group II MAbs did not cross-block CS-E, in contrast to group III MAbs, we proposed that the shared epitope residues of peptide 58 could not be responsible for the group III MAbs cross-blocking of CS-E (amino acids H95, W96, N97, Y101, S103 and E106) [9]. K108, however, is unique to the group III epitope and is involved in CS-E binding, based on our docking result (see below). Therefore, we hypothesized that residue K108 is responsible for cross-blocking differences between group II and group III. Other intersecting D8 residues are necessarily involved in the case of group III MAbs that induce high levels of CS-E blocking, such as EE11 (60–80% cross-blocking; Fig. 1B, C).

**Figure 2:**
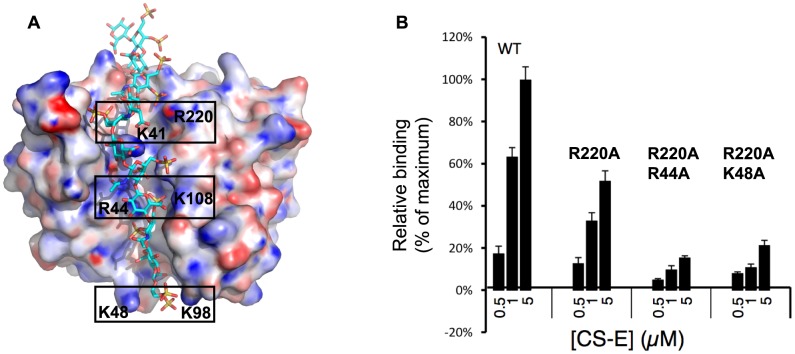
Mapping of the CS-E binding site on vaccinia D8 ectodomain. **A.** Docking of CS-E dodecasaccharide to D8. Framed regions highlight regularly spaced, positively charged residue pairs K41/R220, R44/K108, and K48/K98, which are predicted to interact with negatively charged sulfate moieties of CS-E. **B.** Mapping of CS-E binding site. Mutation R220A led to a ∼50% decrease in CS-E binding compared to wt, while CS-E binding to the double mutants R220/R44 and R220/K48 was almost fully abrogated, corroborating the CS-E docking model. Data were averaged from three experiments.

These CS-E binding data led us to refine our previous docking results by using a dodecasaccharide fragment of CS-E, instead of the previously used CS-A [8]. In the docked model, each sulfate group is found in the vicinity of the charged D8 residue pairs K48/K98, R44/K108, and K41/R220, delineating the crevice, which corroborates the high specificity of CS-E over CS-A that was used for docking prior to our knowledge of the exact ligand (Fig. 2A). In effect, CS-A bears only one sulfate group, on 4′-hydroxyl of GalNAc, while CS-E has an additional sulfate on the 6′-hydroxyl group. Hence, docking data converged with the experimental definition of the ligand, since it pointed to the aforementioned positively charged residual pairs that are probably necessary to form salt bridges with both sulfate moieties. Alanine substitutions of residues lining the crevice led to moderate (R220A) or severe (R220A/R44A, and R220A/K48A) reductions in CS-E binding (Fig. 2B). This suggests that D8 binding to CS-E is likely mediated by a network of electrostatic interactions that form pairs on opposing sides of the entire D8 crevice, involving residues K41, R44, K48, K98 and K108 (Fig. 2A). In the D8/LA5-Fab complex [MAb of group IV, pdb code 4ETQ], we observed that only two of these residue pairs (K41/R220 and R44/K108) are part of the epitope. However, LA5 fully blocks CS-E binding of D8, even without K48/K98 coverage.

### Identification of anti-D8 MAb epitopes by EM

**Figure 3:**
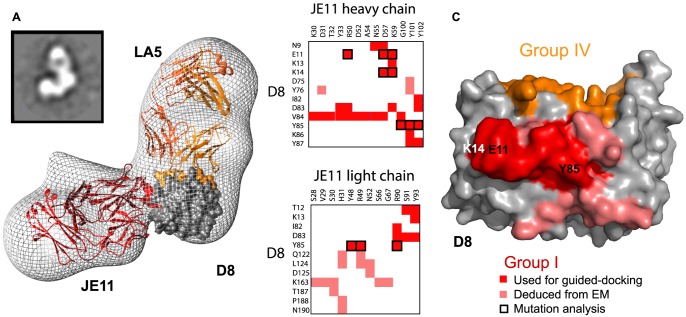
Group I (JE11) footprint. **A.** EM reconstruction of the D8 monomer in complex with Fab's JE11 (group I) and LA5 (group IV) at 24 Å resolution. Projection Matching and Fourier Shell Correlation (FSC) are shown in figure S5. Top left inset shows one of the class-averages used for building the map. EM density is shown in gray mesh. D8 monomer crystal structure is represented as a grey surface except for epitope footprints that follow the same color code as [9]: group I (JE11): red; group IV (LA5): orange. Actual Fab chains also follow this color code. **B.** Summary of JE11 (group I) contacts. D8 residues in red belong to the initial definition of group I epitope, assessed by DXMS. Salmon-colored residues complete the definition of group I conformational epitope. Black bold-contours highlight residues previously picked for mutation analysis [9]. **C.** Footprint of completed JE11 epitope. Red and salmon footprints evidence initial and additional epitope residues. Despite being juxtaposed to each other, group IV (LA5) and group I (JE11) footprints do not intersect. Black labels inform on residues resulting in a loss of MAb/Ag affinity upon mutation to alanine, while mutated residues in white did not lead to any relevant change in binding.

**Figure 4:**
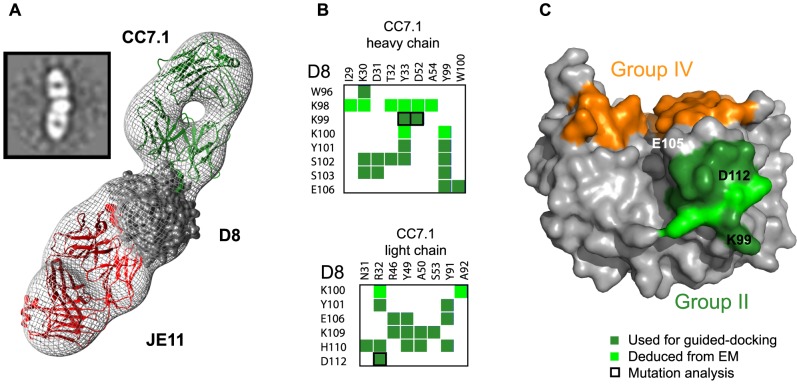
Group II (CC7.1) footprint. **A.** EM reconstruction of the D8 monomer in complex with Fabs JE11 (group I) and CC7.1 (group II) at 21 Å resolution. See figure legend 3 for general description. Projection Matching and Fourier Shell Correlation (FSC) are shown in figure S6. Epitope footprints follow the same color code as [9]: group I (JE11): red; group II (CC7.1): green. **B.** Summary of CC7.1 (group II) contacts. D8 residues colored in forest green belong to the initial definition of group I epitope, assessed by alanine scanning. Lighter green residues complete the definition of group II epitope. **C.** Footprint of completed CC7.1 epitope. Forest green and light green footprints evidence initial and current epitope definitions. Group IV (LA5) footprint in orange does not intersect with group II (CC7.1) epitope.

Determining the full epitope for group II and III MAbs is necessary to explain the wide spread in CS-E blocking ability (10–80%) observed between group II and group III MAbs. To address this question, we used negative stain single particle D8:Fab EM reconstructions. To map the relative positions of the different antibodies on D8, we used Fabs from two different groups, simultaneously bound to D8. JE11-Fab (group I) was included as a reference in all subsequent ternary complexes because it does not cross-block binding of any other MAb groups [9]. We then reconstructed the three-dimensional arrangements by embedding individual atomic models within the low-resolution maps of the multivalent complexes obtained by EM. Docking was guided using previously determined experimental constraints: X-ray crystallography definition of group IV epitope, DXMS definition of group I epitope, and alanine-scan definitions of groups II and III epitopes [9]. Since we had previously determined the high-resolution crystal structure of D8/LA5 (group IV), we first reconstructed the D8/JE11/LA5 complex (Fig. 3). We then reconstructed the D8/JE11/CC7.1 complex, since a definition of the group II epitope was available (Fig. 4). Fitting of those reconstructed atomic models gave best-fit correlation values of 0.9016 and 0.9207, respectively. These two first processes served as an internal proof of concept, illustrating that our experimental epitope definitions agreed with the associated EM maps.

Total BSA for group I (JE11) D8:MAb interface is 1238 Å^2^. EM data suggests the following additional residues for group I epitope, adding to the strict definition previously obtained by DXMS: N9, D75, Y76, Q122, L124, D126, K163, T187, P188, and N190 (Fig. 3B, C). Looking back at the DXMS data [9], we saw that most of the aforementioned residues are in D8 regions where deuterium (H^2^) exchange decreased upon complex formation, but was weak or inconsistent. Most of these additional residues interact with the JE11 light chain (Fig. 3B, C).

The group II (CC7.1) MAb:D8 interface has a total BSA of only 851 Å^2^, which correlates with the linear nature of group II epitope. Residues 91–110 correspond to a protruding region at the surface of D8. Only minor differences were observed for the group II epitope when comparing EM data to group II alanine scanning definition: EM defined additional residues K98 and K100 as part of the epitope (Fig. 4). However, K98A and K100A showed no reduced binding to MAb CC7.1 [9], suggesting that both residues do not contribute greatly to antibody binding.

### Group III (EE11) epitope definition by EM

**Figure 5:**
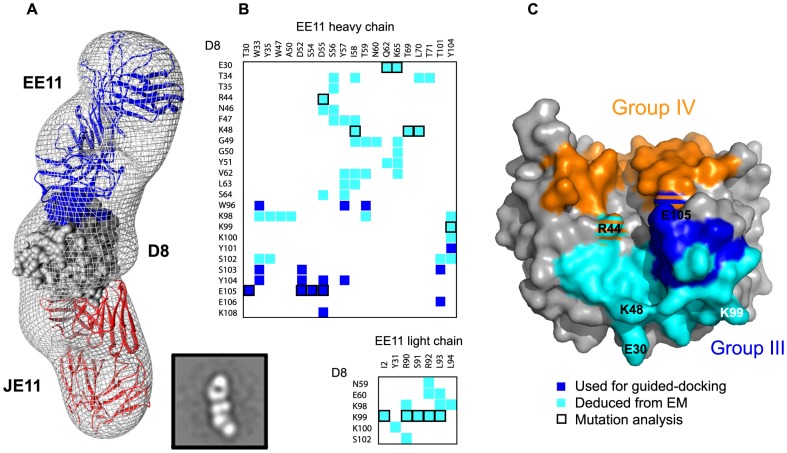
Group III (EE11) footprint. **A.** EM reconstruction of the D8 monomer in complex with Fabs JE11 (group I) and EE11 (group III) at 22 Å resolution. See figure legend 3 for general description. Projection Matching and Fourier Shell Correlation (FSC) is shown in figure S7. Epitope footprints follow the same color code as [9]: group I (JE11): red; group III (EE11): blue. Actual Fab chains follow the same color code. **B.** Summary of EE11 (group III) contacts. D8 residues colored in blue belong to the initial definition of group III epitope, assessed by alanine scanning. Cyan residues complete the definition of group III epitope. **C.** Footprint of completed EE11 epitope. The initial definition obtained by alanine scanning and PMA is depicted in blue and the current definition deduced from the EM particle reconstruction is in cyan. Group IV (LA5) footprint in orange does intersect with group III (EE11) epitope at residues R44 and K108 (orange/cyan or orange/blue stripes).

We used EE11-Fab to build a group III ternary complex for which we did not have a full definition (Fig. 5). Model-to-map correlation for this complex was 0.9115. The total EE11:D8 BSA was 1710 Å^2^, which is larger than group I and II epitopes but smaller than group IV (2434 Å^2^). A total of twenty-six D8 residues interact with EE11 MAb. Six D8 residues interact with the light chain (LC) and twenty-four with the heavy chain (HC) (Fig. 5B, C). Novel D8 contacts are E30, T34, T35, **R44**, N46, F47, **K48**, G49, G50, Y51, N59, E60, V62, L63, S64 and additional peptide 58 residues **K98**, K99, K100 and S102, with residues involved in CS-E binding indicated in bold. Group IV (LA5) footprint (in orange) intersects with the group III (EE11) epitope at residues R44 and K108 (Fig. 5C).

**Figure 6:**
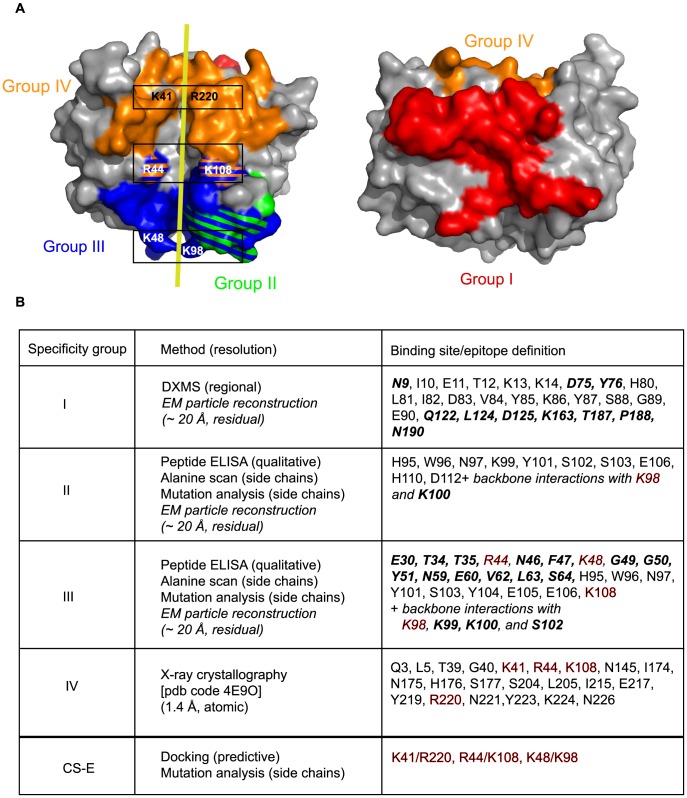
Summary of D8 murine epitome and CS-E binding site. **A.** Updated footprint of groups I (red), II (green), III (blue) and IV (orange) are represented. The yellow line reminds the CS-E path between positively charged residue pairs (black frames). **B.** Summary of D8 epitope residues for all VACV anti-D8 murine MAbs of the four epitope groups. Resolution depends on the method used for a specific assessment. Newly-defined epitope residues are highlighted in bold italic. Residues in red are important for both CS-E, and group III and IV MAb binding.

The resolution of the EM maps obtained with negative staining provides an accurate epitope definition of group III but not at atomic resolution. In order to validate this newly defined interface, we picked three seemingly critical residues for alanine scanning mutagenesis, two of which are involved in CS-E binding (E30, **R44**, **K48**). A 3-fold decrease in affinity was observed for D8 E30A (Fig. S2). However, this was likely due to suboptimal folding of D8 E30A, as the control antibody (JE11) also showed reduced binding (9-fold). In both cases, only the association phase was affected. This suggested that E30 does not contribute greatly to EE11 binding. We observed an almost 10-fold decrease in EE11 affinity with D8 R220A/R44A and R220A/K48A mutants compared to either wild-type (wt) D8 or R220A D8, suggesting both R44 and K48 are important residues for EE11 binding (Fig. S2). Both mutants bound normally to control antibodies. Figure 6 summarizes the details of and the techniques used to identify the complete murine D8 epitome.

Most of the CS-E binding residues of D8 are targeted by the HC of EE11 (K41, R44, K98, and K108), while the EE11 LC has a single contact (K98; Fig. 5B). Hence, our model is compatible with the general assumption that the HC frequently drives most Ag/MAb interactions [12]. However, the EM model does not corroborate our hypothesis that LC differences were responsible for the CS-E blocking differences within group III.

### D8 oligomerization is mediated through the C-terminal domain

**Figure 7:**
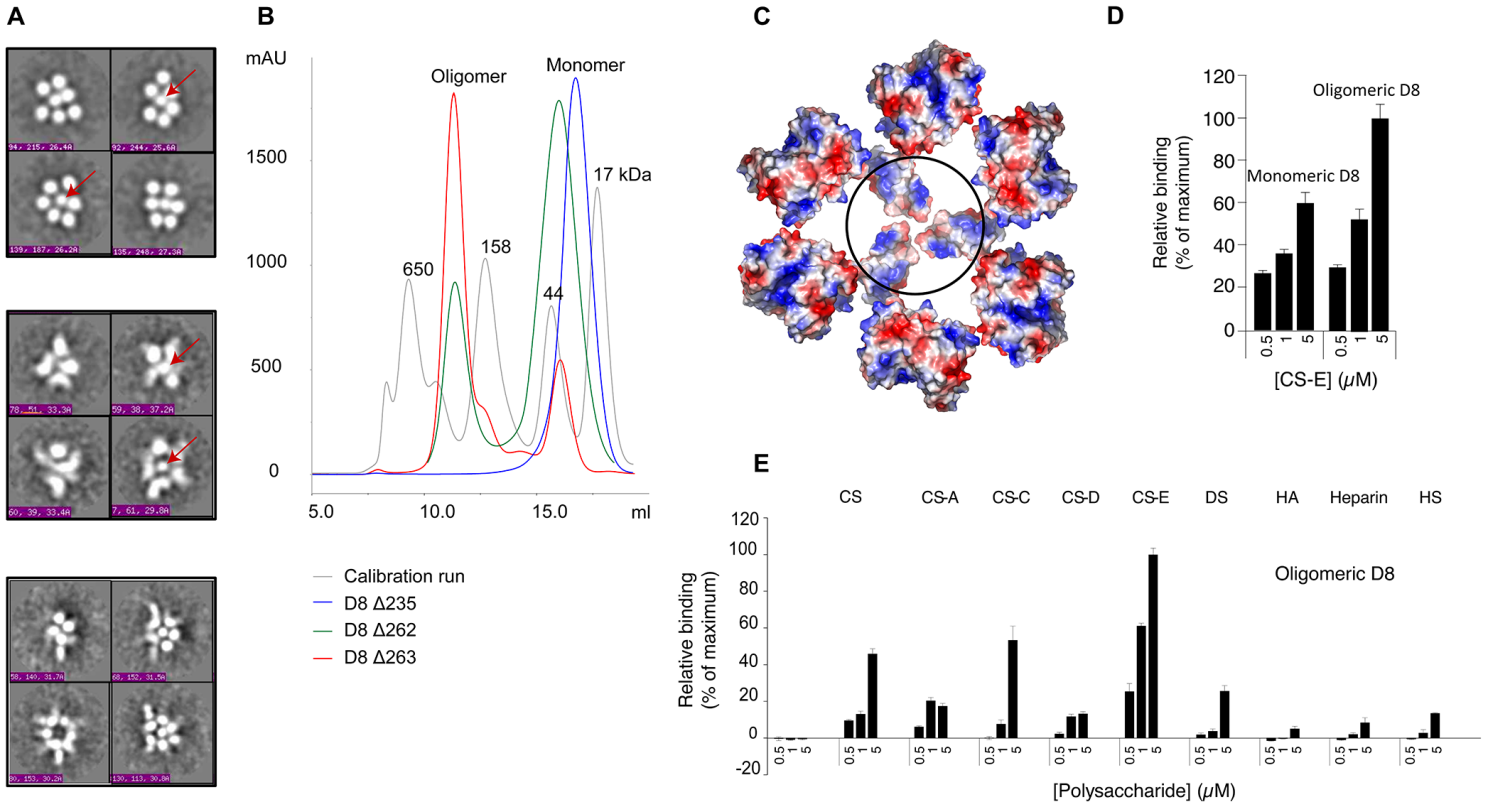
D8 oligomeric interface is secluded to the C-terminal region 235–262. **A.** From top to bottom: selected class averages of (i) unliganded D8 oligomer, (ii) oligomeric D8+JE11-Fab, and (iii) oligomeric D8+JE11-Fab+LA5-Fab. Despite the monodispersity of unliganded D8 oligomeric sample, particles showed a varying number of drupelets, because of their orientation on the EM grid. The class averages with the highest number of drupelets always show six drupelets surrounding a central one (red arrows). **B.** SEC profiles of recombinant D8 Δ235, Δ262, and Δ263 suggest that D8 oligomerises through the C-terminal domain. SEC markers as grey curve with MW given in kDa. **C.** Putative D8 hexameric model based on EM data and SEC-MALS using biochemical constraints relative to the dimer and oligomer interfaces. The black circle highlights the putative seventh and central drupelet, arising from all six SU C-terminal extremities, converging toward the IMV envelope. **D.** CS-E microarray indicating that D8 hexamer (0.33 µM) binds more effectively to CS-E compared to 6-times molar excess of D8 monomer (2 µM) **E.** GAG microarray obtained with D8 oligomer (0.1 µM). D8 oligomer binds CS-E with higher affinity than the monomer, and also weakly binds to other CS species but not to DS, HA, heparin and HS. Micro array binding experiment was performed in triplicate, and the data represent the average of 10 spots per concentration averaged from the three experiments (±SEM, error bars).

For structural studies, we prepared monomeric D8 that either only contains the CAH domain (residues 1–234) or lacks the distal C-terminal cysteine (C262, residues 1–261). However, we have previously shown that the full D8 ectodomain forms a disulfide-linked dimer (through C262) that further associates non-covalently to form an oligomer [8]. Despite oligomeric D8 having an molecular weight that was considerably larger than the 158 kDa MW marker on SEC, we had previously described the D8 oligomeric state as tetrameric, based on D8 migration during native gel electrophoresis [8]. However, size exclusion chromatography with inline multi-angle light scattering (SEC-MALS), assigned an average MW of 228 kDa for the D8 oligomer compared to 42 kDa obtained for the D8 monomer, using two different SEC resins (Fig. S3). The SEC-MALS data indicate a hexameric D8 arrangement. EM class averages of the D8 oligomer revealed no more than 7 drupelets, with 6 drupelets surrounding a central drupelet that appears of lesser intensity (Figs. 7A, S3 and S4). While a D8 heptamer remained a possibility, our SEC data coupled to non-reducing SDS-PAGE suggested that the D8 oligomer is formed by en even number of D8 monomers. When subjected to SEC both D8 oligomers, either lacking (1–261) or containing (1–262) the C-terminal cysteine, eluted at the identical volume (Fig. 7). When the D8 oligomers were subjected to non-reducing SDS PAGE, no D8 monomer, but only disulfide-linked D8 dimers were observed [8]. Therefore, only an even numbered D8 oligomer, such as a hexamer appears possible, as an octamer would not be supported by the SEC-MALS data. We speculate that the 7^th^ and central drupelet is not a result of heptameric D8 but rather formed by the convergence of the C-terminal extremities (235–262) of all six D8 subunits (SUs). We confirmed the monodispersity of the sample by size SEC-MALS (Fig. S3), which suggested that 2D class averages are of a single species, and most likely represent different orientations of the hexamer (Fig. S4A). However, obtaining three-dimensional maps of the D8 hexamer was problematic due to apparent conformational flexibility of D8. In fact, the CAH fold domains do not adopt a uniform arrangement in the different hexameric D8 class averages of figure 7A, but are instead placed haphazardly around the central drupelet, likely due to flexibility of the connecting linker to the C-terminal domain. The C-terminal domain was also disordered in the crystal structure of D8 [8]. Hence, multiple conformations of the D8 hexamer prevent the reconstruction of a three-dimensional model via EM. This flexibility also suggests that CAH fold domains do not participate in the oligomeric interface and correlates with an apparent higher MW for the D8 hexamer (MW of 228 kDa compared to theoretical 192 kDa).

Until now, no function was assigned to the C-terminal domain of D8. To test our hypothesis that this domain mediates oligomerization, we compared SEC elution profiles of three D8 constructs of different lengths. D8 Δ262 is mostly monomeric when cells are grown at 37°C, and has a surprisingly higher ratio of hexamer when grown at lower temperature (30°C). This construct was used to solve the structure of the D8 monomer/LA5-Fab complex. In order to favor crystallization, we intentionally did not include unique cysteine 262. When including cysteine 262 (D8Δ263 construct), we observed a higher ratio of oligomer vs. monomer (Fig. 7B). The third construct, D8Δ235, contains only the CAH fold domain. D8Δ235 was solely monomeric, which confirmed the mapping of the D8 oligomeric interface to the C-terminal region. These findings now assign a function to this domain. A model of the D8 hexameric arrangement is shown in figure 7C. In this model, both sides of a D8 monomer are in close proximity to the two neighboring D8 monomers. Those are also the D8 surfaces that are not targeted by any of the murine antibodies, likely due to inaccessibility, further validating the hexameric “ring-like” model.

Finally we have assessed the role of D8 oligomerization in CS binding, using the GAG microarray binding assay (Fig. 7D). A 40% increase in CS-E binding to oligomeric D8 compared to the monomer was observed, correcting for the six times molar excess of D8 monomer. Therefore, oligomerization increases binding avidity. In addition, and maybe more importantly, slight binding to other CS species could also be detected. This was especially true for CS-C, and was also observed for CS-A to a low degree. Together, with more optimal CS binding to host cells, we speculate that D8 oligomerization increases viral avidity to CS-E but also to other CS species. This may improve viral adhesion to cells expressing low levels of surface CS-E, or heterogeneous populations of CS.

## Discussion

In this study, we have completed the determination of the murine D8 antibody epitome, proposing a novel definition of the group III epitope. Involvement of K108 explains the competition between group III and IV MAbs. These data also explain why group III MAbs block CS-E binding when group II does not. Sequence analysis, together with the observed overlap of MAb group II and III epitopes lead us to consider group II as a sub-group of group III. The group II epitope is a linear, minimal version of a larger epitope space that includes group III conformational epitopes. Group III MAbs have increased CS-E cross-blocking abilities as their epitopes diverge from linear to conformational, as well as increase in size. As a result, CS-E cross-blocking ability of these MAbs increased. Because EE11 includes the maximal number of CS-E binding site residues that group III MAbs may target (R44, K48, K98, K108), we believe that the EE11 cross-blocking ability represents group III's CS-E blocking maximum. In contrast, glycan microarray data place BH7 at the bottom end of group III MAb cross-blocking, and therefore the BH7 epitope may (i) be narrower than EE11, and (ii) include at least CS-E contacting residue K108. Group III MAbs display cross-blocking levels between those of group II and IV. We conclude that group III MAbs nuance their CS-E blocking abilities by including additional CS-E binding residues to their epitope (R44, K48, and/or K98). One can use CS-E binding data to predict the size of their respective epitopes. CS-E blocking abilities of all anti-D8 MAbs is hierarchized as follows: LA5>EE11≥JF11∼JE10>BG9>JA11>BH7>CC7>JE11. Total BSAs of the different groups' epitopes can be ranked as follows: IV (LA5: 2434 Å^2^) [8] >III (EE11: 1710 Å^2^)>I (JE11: 1238 Å^2^)>II (CC7: 851 Å^2^). Consequently, we predict epitopes of group III JF11 and JE10 to target a region similar to EE11 on D8's surface that most likely includes all four possible CS-E binding residues within this epitope, since all three MAbs have maximal CS-E blocking abilities within group III. JA11 is at the other end of the CS-E blocking range, and therefore its epitope might be close to the minimal definition for group III. Lastly, BG9 has an intermediate blocking ability of 50%, suggesting it targets an intermediate-sized epitope that includes K108 and no more than two other positively charged residues, such as R44, K48, and/or K98.

The importance of D8/CS-E adhesion for subsequent VACV infection remains unclear. It is conceivable that the CS-E ligand may be restricted to small pools of target cells in certain organs [13], [14]. *In vivo* infection models have not been discriminative of cells based on their surface sugar profiles. In addition, it is possible that there are species differences in glycosylation between mouse and man that alter poxvirus pathogenesis. Alternatively, the sugar binding properties of D8, H3, and A27 may have evolved to be redundant or combinatorial with each other. The highly sulfated CS-E type has been shown to bind to heparin binding growth factors midkine (MK), pleiotropin (PTN), heparin-binding epidermal growth factor-like growth factor (HB-EGF), FGF-16, and FGF-18. As many of these growth factors are expressed in the mammalian brain, it was proposed that CS-E and CS proteoglycans (CSPGs) are critical to the development of the brain and central nervous system [15]. Subsequent work using an antibody specific for the CS-E disaccharide revealed its presence in the developing mouse brain. The associated strong expression of the gene for GalNAc4S-6ST transferase confirmed that CS-E chains are critical in brain development, with the implication that CS-E chains participate in neurogenesis, axon guidance, and/or neuronal survival [16].

Interestingly, a VACV D8 knockout was unable to infect the rat brain, suggesting that D8 function is connected to neural tissues [17]. An *ex* or *in vivo* experiment with cells displaying the CS-E+/HP-/HS- phenotype is essential to test the hypothesis that orthopoxviruses may use CS-E as a selective infection route. In effect, an infectious route relying on a non-ubiquitous GAG such as CS-E may be strategically effective, since the virus would avoid binding to most cells and therefore more efficiently target the desired cell types for infection. In addition, perhaps binding CS-E is a strategy for orthopoxviruses to build dormant pools of virus, or to travel long distances within axons of neural cells, for example. However, such subtle mechanisms may be hidden under the main infection routes that involve binding to heparin sulfate by the viral attachment proteins A27 and H3 [4], [5].

## Materials and Methods

### D8 cloning

D8 Δ262 construct (amino acids 1–261, lacking Cys 262) was engineered and prepared as reported previously [8]. D8Δ263 (containing Cys 262) protein expression vector was designed by modification of the pET-22b(+):: D8Δ262 expression vector through site-directed insertion of the C262 mutation using the QuikChange II Site-Directed Mutagenesis Kit (Agilent) with primers 5′-TCCGATTTGAGAGAGACATGCCTCGAGCACCACCACCAC-3′ and 5′-GTGGTGGTGGTGCTCGAGGCATGTCTCTCTCAAATCGGA-3′.

The D8Δ235 construct contains only the carbonic anhydrase domain of D8 and was obtained by overlapping PCR using the following primers to amplify the D8Δ235 sequence from VACV ACAM2000 genomic DNA with the Accuprime Pfx PCR Kit (Invitrogen). Primer 1: 5′-CTTTAAGAAGGAGATATACATATG CAACAACTATCTCCTATT-3′, Primer 2: 5′-GTGGTGGTGCTCGAGAGAATAATATACTTCTGTGTCATC-3′ [18]. A 134∶1 molar ratio of gel-purified PCR product to pET22b(+) was mixed to 1 uL KOD HiFi Polymerase, 1 µL 10× KOD Buffer, 1 µL 8 mM dNTPs, 1 µL 10 mM MgCl_2_ in a final 10 µL reaction volume (Toyobo). The following thermocycler protocol was then used: 98°C for 2 min, 20 cycles of 98°C for 30 s, 55°C for 30 s and 72°C for 8 min, followed by final extension at 72°C for 20 min. Template DNA was digested with DpnI for 1 hr at 37°C and used to transform DH5α cells for plating on LB Agar with ampicillin. DNA was isolated from 5 mL cultures of single DH5α colonies by Miniprep (Fermentas) and successful cloning was confirmed by sequencing (Retrogen).

For mapping of the CS-E binding site, D8 Δ262 R220A, R44/R220, and K48/R220 were expressed and purified as reported previously [9] with addition of a final size exclusion chromatography (SEC) step to isolate monomeric fractions.

### Protein expression and purification

BL21-CodonPlus(DE3)-RIL competent cells (Agilent) were transformed with one of the D8 expression vectors and grown in LB media with 1 mM Ampicillin at 37°C until OD_600_ ∼0.6. Protein expression was then induced with 1 mM IPTG for 4 hrs at 37°C, while shaking at 230 rpm. Cells were pelleted and resuspended in lysis buffer containing 100 mM Tris pH 8.0, 300 mM NaCl, 0.5 mM EDTA, 20 mM Imidazole, 0.2 mM PMSF and lysed under 20000 psi pressure using a microfluidizer (Microfluidics). Cell lysate was clarified at 50,000 g for 20 min. Supernatant was loaded onto 5 mL Ni-NTA column (His-Trap, GE). Bound D8 protein was eluted with 20 mM Tris pH 8.0, 300 mM NaCl, 200 mM Imidazole. After overnight dialysis against 20 mM Tris pH 8.0, 200 mM NaCl, the sample was concentrated and subjected to SEC using a Superdex 200 10/300GL column (GE) in the same buffer. The monomeric peak with V_E_≅16.5 mL and oligomeric peak with V_E_≅11.7 mL were collected in separate fractions.

### Glycosaminoglycan microarray assay

Microarrays containing natural GAGs enriched in CS-A, CS-C, CS-D, and CS-E (Seikagaku Corp., Tokyo, Japan), dermatan sulfate (DS; Sigma-Aldrich, St. Louis, MO), hyaluronic acid (HA; Sigma-Aldrich, St. Louis, MO), heparin (Hep; Neoparin, Alameda, CA), heparan sulfate (HS; Sigma-Aldrich, St. Louis, MO), or chondroitin sulfate (CS; Sigma-Aldrich, St. Louis, MO) were printed on poly-DL-lysine-coated glass surfaces as described previously [19], [20]. Arrays were blocked with 10% FBS in 1× PBS with gentle rocking at room temperature for 1 h, followed by a brief rinse with 1× PBS. For binding and mapping experiments, monomeric D8, monomeric D8 mutants R220A, R44A/R220A, K48A/R220A, and oligomeric D8 were diluted to 2 µM (monomeric) and 0.33 µM (oligomeric) in 1× PBS containing 1% BSA; 100 µl was spotted on the microarrays and incubated at room temperature for 3 h. For antibody blocking experiments, 0.1 µM oligomeric D8 was incubated with 1 µM MAb (group I: JE11; II: CC7; III: BH7; BG9; EE11; JA11; JE10; IV: LA5) or alone for 1 h at room temperature; 100 µl was spotted on the microarrays and incubated at room temperature for 3 h. Microarrays were rinsed briefly three times with 1× PBS and incubated with 1∶200 rabbit anti-6-His (Bethyl Laboratories, Montgomery, TX) for 1 h with gentle rocking, rinsed briefly three times with 1× PBS, followed by 1∶5,000 Cy3-conjugated goat anti-rabbit IgG antibody (Jackson ImmunoResearch, West Grove, PA) for 1 h in the dark with gentle rocking. The microarrays were then washed (3 times of 1× PBS and 2 times of de-ionized water), dried under a stream of air, and scanned at 532 nm using a GenePix 5000a scanner. Fluorescence quantification was performed using GenePix 6.0 software (Molecular Devices, Sunnyvale, CA). CS-E cross-blocking abilities of the four specificity group MAb representatives, and of group III MAbs were calculated for a CS-E concentration of 5 µM.

### D8/CS-E docking

CS-E ligand was built by fusing two CS-A hexasaccharides extracted from CS/cathepsin K complex structure [pdb code 3C9E] [21]. Additional sulfate groups were added to the ligand at position 6′ of N-acetyl-beta-D-galactosamine-4-sulfate, and ligand geometry was regularized and energy-minimized with PRODRG [22]. Docking was performed with Autodock Vina using a D8 pdbqt-formatted structure file [23] along with chondroitin 4,6-sulfate (CS-E) dodecasaccharide as ligand. In the original D8 coordinate file [pdb code 4E9O] [8], K48 Nξ interacts with water 334, and water 326 of adjacent symmetry mate, and is pointing outward, suggesting it is not constrained in solution. In order to allow optimal docking of CS-E, side chain of residue K48 was moved away from K98 side chain (Coot, rotamer 5, 4% likelihood, Chi1 = −177°). Grid box dimensions (x, y, z = 120, 48, 54) and center (x, y, z = 18.169, −0.779, −10.045) were defined based on our early routines [8] in order to accommodate the new, larger ligand.

### Fab preparation

IgG's were subjected to papain digestion to produce Fabs. Conditions for digestion were as follows: EE11-IgG2a was digested with 2% w/w activated papain in 50 mM NaOAc pH 5.5 reaction buffer at 37°C over 3 hrs; JE11 and LA5-IgG2a were digested with 2% w/w activated papain in 50 mM NaOAc pH 5.5 reaction buffer at 37°C over 4 hrs; CC7.1 (IgG1) was digested with 2% w/w activated papain in 100 mM Tris pH 7.0 reaction buffer with 10 mM cysteine at 37°C over 2 hrs. Papain digestion was terminated by addition of 20 mM iodoacetamide. EE11, JE11, and LA5-Fab containing samples were then dialyzed overnight in 5 L PBS pH 8.0. Samples were then passed through 1 mL FF Protein A column (GE) in PBS pH 8.0 binding buffer and Fab was collected in the flow-through. Fab was further concentrated for subsequent purification by SEC using a Superdex 200 10/300GL column (GE). Purified monomeric Fab peaks (V_E_≅16 mL) were collected for complex preparation. The CC7.1 Fab containing sample was buffer exchanged against 3 M NaCl, 1.5 M Glycine pH 8.9 for Protein A affinity purification. Flow-through was dialyzed overnight against 5 L 20 mM Tris pH 8.0, 200 mM NaCl and subjected to SEC using a Superdex 200 16/60HR column (GE). Fractions containing monomeric Fab were collected for complex preparation (V_E_≅86.4 mL).

### Complex preparation

Monomeric D8 protein was used to prepare the D8 ternary and quaternary complexes with two different specificity groups Fab molecules (D8/JE11/LA5, D8/JE11/LA5/CC7.1, and D8/JE11/EE11). The D8/LA5 crystal structure was used to position LA5-Fab in the EM map, while subsequently JE11 was used as a position marker for single particle EM reconstruction, since it does not cross-block binding of any of the other specificity group MAbs. The D8 Δ263 oligomeric protein was used to assess the physiological D8 oligomerization state with or without Fab decoration.

#### D8/JE11/LA5

D8Δ262 monomer+JE11-Fab complex was prepared by mixing 20% molar excess of D8Δ262 monomer to purified JE11-Fab at low concentration (∼0.2 mg/mL). Sample was then incubated on ice for 5 minutes, concentrated, and loaded onto a Superdex 200 10/300 SEC (GE). The D8Δ262 monomer+JE11-Fab complex (∼82 kDa) was pooled and mixed with LA5-Fab at equimolar ratio. The ternary complex sample was concentrated and purified by SEC (Superdex 200 10/300) as a ∼152 kDa protein complex.

#### D8/JE11/LA5/CC7.1

This complex was prepared as described above for D8Δ262 monomer+LA5-Fab+JE11-Fab, with the final addition of CC7.1-Fab at equimolar amounts for a final SEC step, where fractions corresponding to D8Δ262 monomer+LA5-Fab+JE11-Fab+CC7.1-Fab quaternary complex were obtained upon SEC as a ∼200 kDa complex. Only few class-averages pertained to the quaternary complex, and, therefore, this sample lead only to 3D-reconstruction of the ternary complex D8/JE11/CC7.1.

#### D8/JE11/EE11

The first step for preparing this complex is similar to the ones mentioned above: JE11-Fab was added to 20% molar excess of D8Δ235 monomer initially, followed by the addition of a 20% molar excess of EE11-Fab to the purified secondary complex pool for final SEC (Superdex 200 10/300). All complexes were freshly SEC-purified prior to EM analysis, and existence of the complex was validated by observation of a proper shift of elution volume upon complex formation, and SDS-PAGE analysis (Fig. S8).

### Electron microscopy

Various complexes were prepared with either monomeric D8, monomeric D8 together with the Fabs from either JE11 and CC7.1, or JE11 and EE11, or JE11 and LA5, or oligomeric D8 either unliganded, or in complex with LA5-Fab, or with both JE11- and LA5-Fabs. Samples were analyzed by negative stain EM. A 3 µL aliquot containing ∼0.05 mg/mL of complex was applied for 15 s onto a carbon-coated 400 Cu mesh grid that had been glow discharged at 20 mA for 30 s, then negatively stained with uranyl formate for 30 s. Data were collected using a FEI Tecnai F20 or T12 electron microscope operating at 120 keV, with an electron dose of ∼27 e^−^/Å^2^ and a magnification of 52,000× that resulted in a pixel size of 2.05 Å at the specimen plane. Images were acquired with a Gatan US4000 CCD or Tietz TemCam-F416 CMOS camera using a nominal defocus of 1000 nm and the Leginon package [24] at 10° tilt increments and up to 50°. The tilts provided additional particle orientations to improve the image reconstructions.

### Data processing and image reconstruction

Particles were picked automatically using DoG Picker and put into a particle stack using the Appion software package [25], [26]. Initial, reference-free, two-dimensional (2D) class averages were calculated using particles binned by two via the Xmipp Clustering 2D Alignment [27] and sorted into classes. Particles corresponding to complexes were selected into a substack and binned by two before another round of reference-free alignment was carried out using the Xmipp Clustering and 2D alignment and IMAGIC software systems [28]. To analyze the interactions of the Fabs (LA5, JE11 and CC7) with D8 monomer, the reference free 2D class averages were examined. An *ab initio* common lines model was calculated from reference-free 2D class averages in EMAN2 [29]. Fab densities were visible after 10 iterations. This model was then refined against raw particles for an additional 89 cycles. EMAN [30] was used for all 3D reconstructions. The resolutions of the final models were determined using a FSC cut-off of 0.5. For the 3D reconstruction of D8 monomer with JE11 plus LA5, a number of 5291 particles were used. For the 3D volume of D8 monomer in complex with JE11 and CC7, a number of 8044 particles were used.

### Fab modeling

Full Fab sequences were reconstructed from our in-house Fv sequenced data. V-, D-, and J-alleles were identified using V-quest of the international immunogenetics system [31]. For example, V- and J-genes IGKV4-57*01 and IGKJ5*01 for κ chain and V-, D-, and J-genes IGHV14-3*02, IGHJ3*01, and IGHD2-3*01 for γ2a chain were assigned for JE11. JE11, CC7.1, and EE11 Fv were modeled using Web Antibody Modeling (WAM) based on the AbM package [32]. Isotype-specific constant region sequences were determined using IMGT database and respective atomic coordinates were appended to the Fv domain after segment fitting.

### Model fitting into the EM densities

First, a guided docking approach was used to obtain putative models of D8/JE11-Fv, D8/CC7-Fv and D8/EE11-Fv: experimental epitope definitions were used as input for Z-DOCK docking ([33]; DXMS for JE11, and alanine scan for CC7 and EE11) while assigning CDR residues as potential contacts in the Ag/MAb interface (Kabat definition) while blocking D8 residues that are not part of the epitope. The first ternary complex to be reconstructed was D8/JE11/LA5; D8/JE11/LA5 EM density was segmented into 5 regions corresponding to every domain (C1, Fv1, D8, Fv2, C2), using the UCSF Chimera ‘Segment map’ function [34]. Coordinates of D8/LA5-Fv were extracted from D8/LA5-Fab structure [pdb code 4ETQ] and fitted into the D8/JE11/LA5 EM density. Best-score fit was selected, after testing possible orientations. Putative ternary complexes were obtained by superimposing D8/JE11-Fv docking models onto the EM map-fitted D8/LA5-Fv complex, using Coot least square fit (LSQ) algorithm and setting D8 as the reference molecule [35]. The resulting ternary complex with the highest map fitting score was selected as the final solution, and conserved domains of LA5 and JE11 were fitted at last in the two remaining segments of the map, located the farthest away from the D8 molecule. In the two consecutive ternary complexes (JE11/D8/CC7 and JE11/D8/EE11), a similar routine was applied by fitting the D8/JE11-Fab model deduced formerly first. For the last model (JE11/D8/EE11), D8 residues outside of the known epitope definition were not blocked. The novel definition of group III epitope was defined by submitting JE11/D8/EE11 model to the Contact Map Analysis server [36]. Buried surface areas (BSA) were calculated using PISA [37].

### Point mutation kinetics analysis by BioLayer Interferometry (BLI)

BLI affinity measurements were determined using the Octet Red 96 (FortéBio Inc., Menlo Park, CA). Anti-mouse Fc Capture (AMC) biosensors were pre-soaked in 1× kinetics buffer containing PBS pH 7.4, 0.01% BSA, 0.002% Tween 20 for 1 hour. Antibodies were immobilized by dipping the AMC biosensors in the antibody solution containing 1× kinetics buffer to a concentration of 3 ug/mL for 300 s. For the baseline step, the tips were soaked in 1× kinetics buffer for 300 s. Association was measured by dipping the biosensors in monomeric D8Δ262 diluted in 1× kinetics buffer to concentrations of 20 nM, 10 nM, 5 nM, 2.5 nM, 1.25 nM, 625 pM, 312.5 pM, and 156.2 pM for 900 s. Dissociation was measured in 1× kinetics buffer for 1200s. All steps are processed at 30°C/1000 rpm. Identical assays were performed for wild-type D8Δ262, and D8Δ262 R220A, D8Δ262 E30A, D8Δ262 R44A/R220A, and D8Δ262 K48A/R220A mutants against antibodies BH7, EE11 and JE11. A negative control antibody A2C7 that targets the VACV antigen A33 was run in parallel to all assays for subtraction of background binding signal. Data was analyzed with the ForteBio Data Analysis Software 7.1 (FortéBio Inc., Menlo Park, CA). Y-axis alignment to baseline step and interstep correction to dissociation step were selected to align curves. Aligned and subtracted curves were processed using Savitzky-Golay Filtering. Curve fittings were derived from application of the 1∶1 binding model with full global fitting.

### Size-Exclusion Chromatography Coupled with Multi-angle Light Scattering (SEC-MALS)

The D8 Δ262 wt monomer and the D8 Δ262 oligomer were loaded onto either a Superose 6 10/30 or Superdex S200 10/30 SEC column (GE Healthcare), which were coupled to an AKTA Avant FPLC system (GE Healthcare) with the following calibrated detection systems: (1) HP1 1050 Hewlett-Packard UV detector; (2) MiniDawn Treos multiangle light scattering (MALS) detector (Wyatt); (3) quasielastic light scattering (QELS) detector (Wyatt); (4) Optilab T-reX refractive index (RI) detector (Wyatt). Analysis of the light scattering data coupled to UV280 and refractive index protein concentration measurements allowed determination of the molar mass of the eluting proteins by using the protein conjugate template in Astra 6 software.

## Supporting Information

Figure S1 **Phylogenic analysis of light and heavy Fv sequences of murine anti-D8 MAbs.** Multiple alignment files of anti-D8 HC and LC CDR sequences were obtained with ClustalW2 [38] and used in Seaview 4.2 to generate the dendrograms [39]. Specificity groups are overlapped onto the dendrograms, using the same color-code as for our previous study [8]. Alignment highlights hypervariable regions H1, H2, H3 and L1, L2, L3 and residues are color coded (orange: GPST; red: HKR; blue: FWY, green: ILMV). Phylogenic analysis of HC-Fv sequences closely correlates with the specificity grouping obtained by cross-blocking ELISA [9]. However, group III LC CDR sequences overlap with those of group I, II and IV MAbs.
(TIF)

Figure S2 **Point mutation kinetics analysis by BioLayer Interferometry.** Real-time binding curves of BH7-, EE11- (both group III), and JE11- (positive control) MAbs to wild-type D8Δ262 and D8Δ262 R220A (positive controls) and indicated mutants to assess the validity of our complementary group III epitope definition. Association (900 s) and Dissociation (1200 s) steps are represented. Curves are colored according to their specific antigen concentration (80, 40, 20, 10, 5, 2.5, 1.25 nM and 625, 312.5, and 156.2 pM). Association rate (k_on_), dissociation rate (k_off_), affinity (K_D_) constant, and fit quality scores are deduced from each set of curves and reported in the bottom table. BLI experiment was performed once
(TIF)

Figure S3 **SEC-MALS of D8 Δ262.** Elution volume and molar mass (MM) for D8 Δ262 oligomer (**A**) and monomer (**B**) obtained using Superose 6. Elution volume and molar mass (MM) for D8 Δ262 oligomer (**C**) and monomer (**D**) obtained using Superdex S200. The horizontal dark line under each peak corresponds to the MM of the eluting sample as determined by SEC-UV/MALS. **E.** Reported SEC-UV/MALS MMs.
(TIF)

Figure S4 **Negative stain EM data of unliganded and Fab-bound D8 hexamer.** 2D class averages of (**A**) unliganded D8 hexamer, (**B**) D8 hexamer bound to JE11-Fab, and (**C**) D8 hexamer bound to JE11- and LA5-Fabs.
(EPS)

Figure S5 **Negative stain of D8 monomer in complex with Fabs LA5 (group IV) and JE11 (group I).** A. Projection matching. B. Fourier Shell Correlation graph.
(EPS)

Figure S6 **Negative stain of D8 monomer in complex with Fabs CC7 (group II) and JE11 (group I).** A. Projection matching. B. Fourier Shell Correlation graph.
(EPS)

Figure S7 **Negative stain of D8 monomer in complex with Fabs EE11 (group III) and JE11 (group I).** A. Projection matching. B. Fourier Shell Correlation graph.
(EPS)

Figure S8 **Preparation of monomeric and oligomeric D8/Fab complexes.** **A, B, and C.** D8-monomer complexes. Associated class averages can be seen in figures S5, S6, and S7 **D.** D8-hexamer complexes. Associated class averages of unliganded and Fab-bound D8 hexamers can be seen in figure S4. All complexes are prepared by performing recursive SEC runs, starting by purifying the D8 monomer or D8 hexamer, and performing subsequent SEC runs for each additional Fab added to the complex being prepared. Curves are colored according to their order in the sequential process (SEC #1: orange, SEC#2: green, SEC #3: red, SEC#4: cyan). MW_app_ markers in kDa are shown for reference (grey curve). Based on the cross-blocking data, we also built the quaternary complex D8/JE11/CC4.1/LA5 (panel a, SEC#4). Existence of this complex is evidenced by the class average in figure S9.
(TIF)

Figure S9 **Quaternary complex D8/JE11/CC7.1/LA5.** **A.** Superimposed maps of D8/JE11/CC7.1 and D8/JE11/EE11 ternary complexes, showing a ∼90° rotation in the way group II and III Fab molecules anchor onto the D8 antigen. **B.** Reconstruction of D8/JE11/CC7.1/LA5 quaternary complex, obtained by overlapping ternary complexes of figures 3 and 4.
(EPS)
