# Supplementary figures and images for: Murine Anti-vaccinia Virus D8 Antibodies Target Different Epitopes and Differ in Their Ability to Block D8 Binding to CS-E
Source: PLoS Pathog. 2014 Dec 4;10(12):e1004495. doi: 10.1371/journal.ppat.1004495 (PMC4256255; doi:10.1371/journal.ppat.1004495)

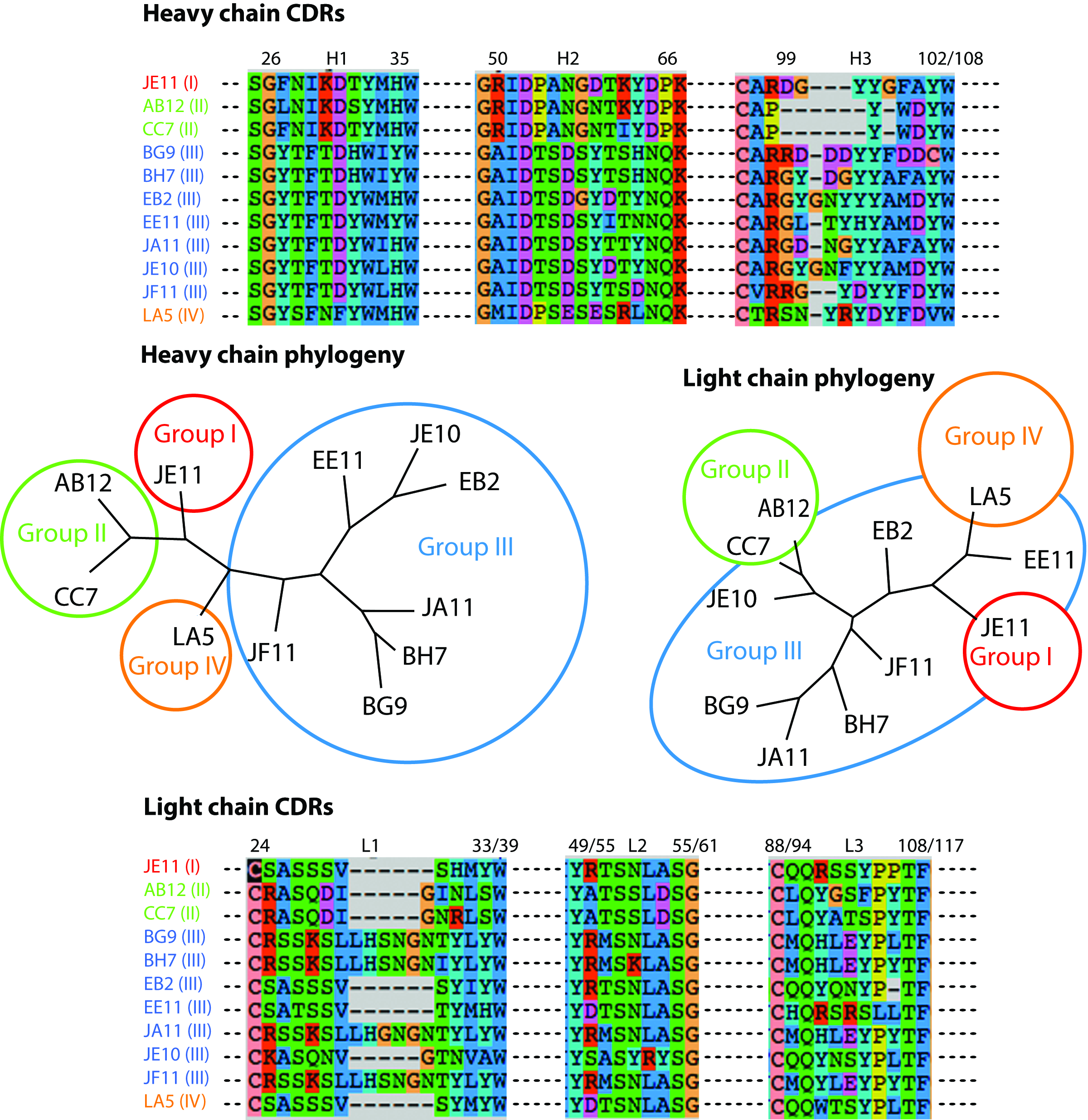

Supplement: Figure S1 — Phylogenic analysis of light and heavy Fv sequences of murine anti-D8 MAbs. Multiple alignment files of anti-D8 HC and LC CDR sequences were obtained with ClustalW2 [38] and used in Seaview 4.2 to generate the dendrograms [39]. Specificity groups are overlapped onto the dendrograms, using the same color-code as for our previous study [8]. Alignment highlights hypervariable regions H1, H2, H3 and L1, L2, L3 and residues are color coded (orange: GPST; red: HKR; blue: FWY, green: ILMV). Phylogenic analysis of HC-Fv sequences closely correlates with the specificity grouping obtained by cross-blocking ELISA [9]. However, group III LC CDR sequences overlap with those of group I, II and IV MAbs. (TIF) [file ppat.1004495.s001.tif]

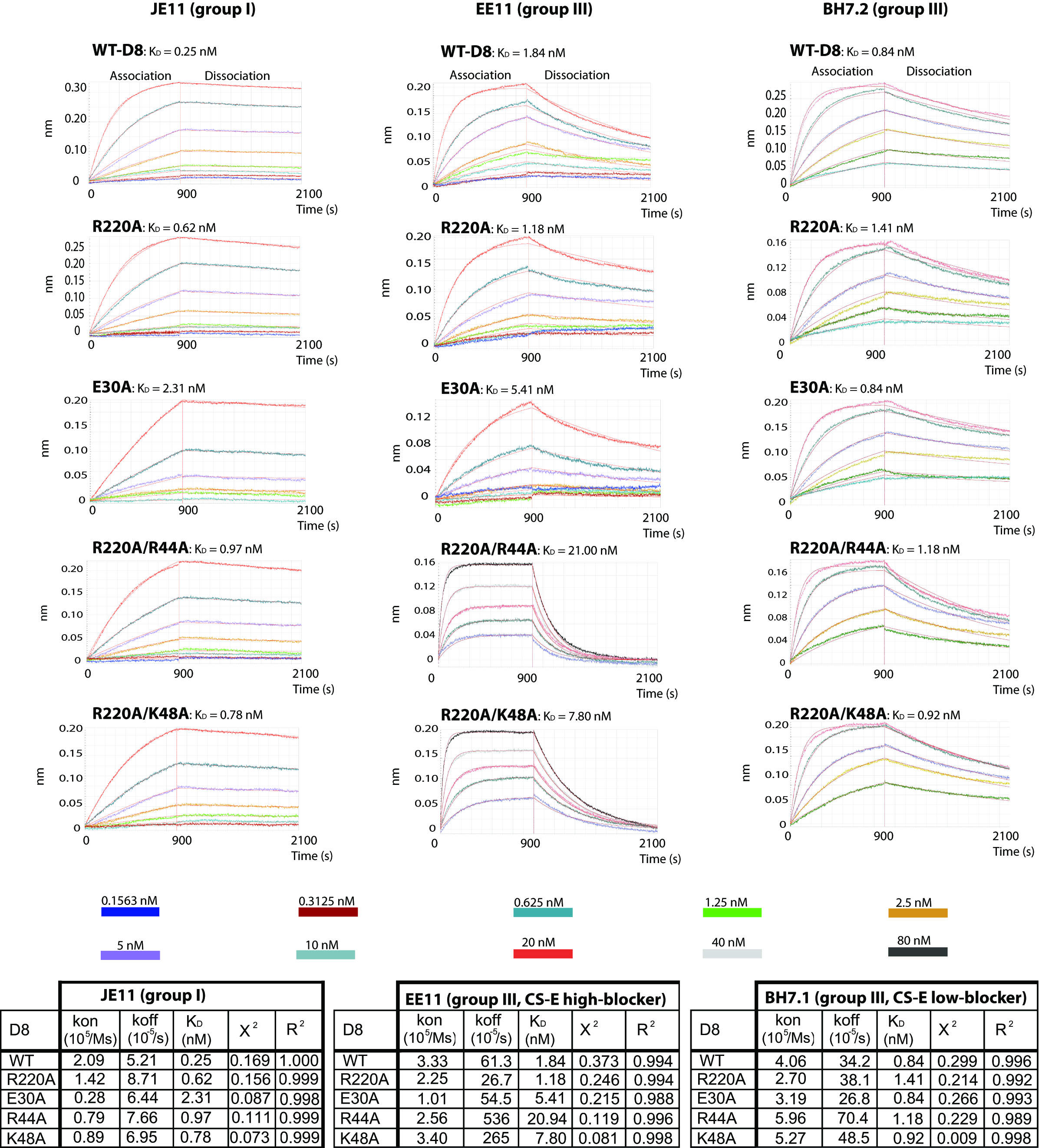

Supplement: Figure S2 — Point mutation kinetics analysis by BioLayer Interferometry. Real-time binding curves of BH7-, EE11- (both group III), and JE11- (positive control) MAbs to wild-type D8Δ262 and D8Δ262 R220A (positive controls) and indicated mutants to assess the validity of our complementary group III epitope definition. Association (900 s) and Dissociation (1200 s) steps are represented. Curves are colored according to their specific antigen concentration (80, 40, 20, 10, 5, 2.5, 1.25 nM and 625, 312.5, and 156.2 pM). Association rate (kon), dissociation rate (koff), affinity (KD) constant, and fit quality scores are deduced from each set of curves and reported in the bottom table. BLI experiment was performed once (TIF) [file ppat.1004495.s002.tif]

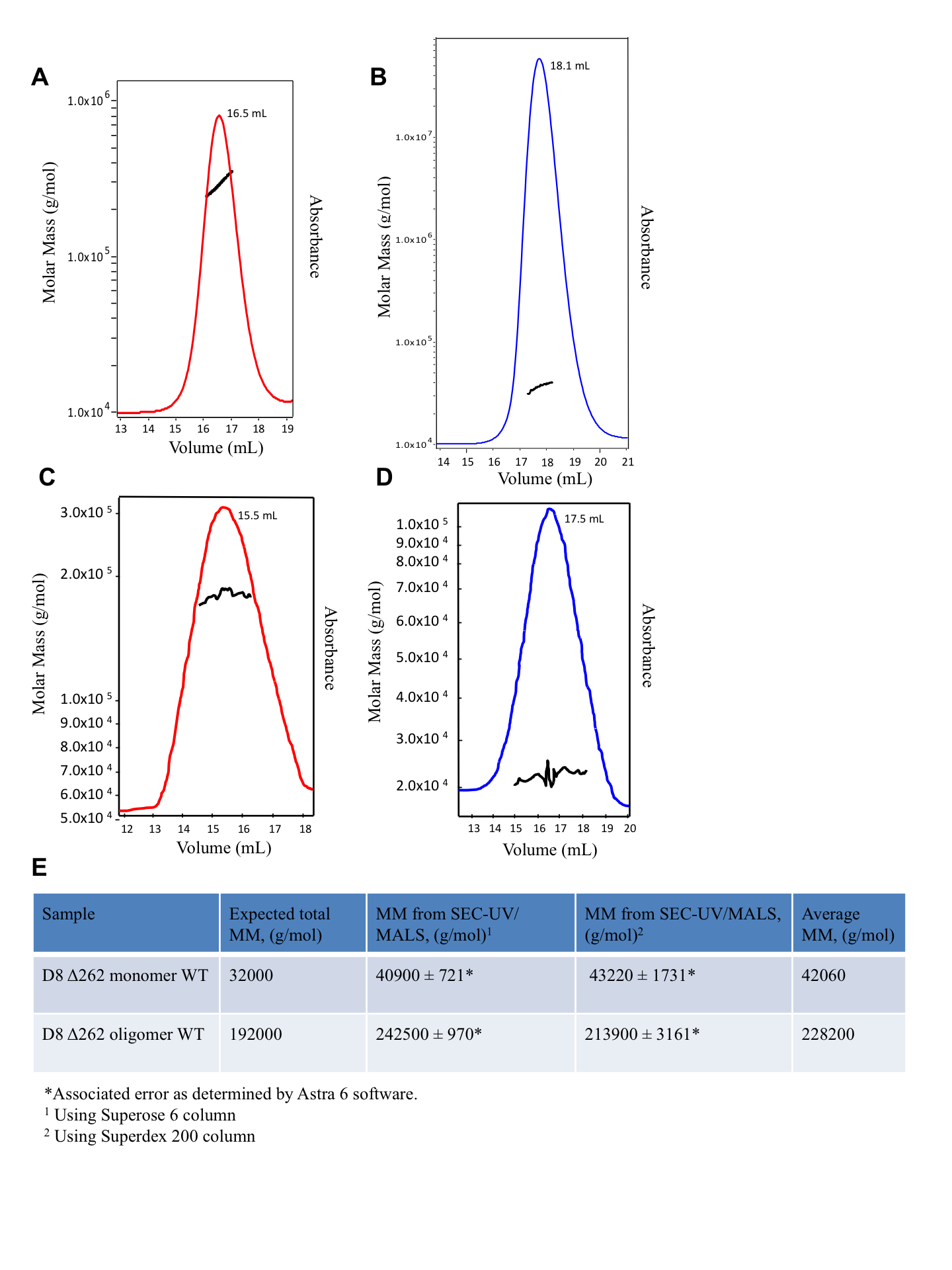

Supplement: Figure S3 — SEC-MALS of D8 Δ262. Elution volume and molar mass (MM) for D8 Δ262 oligomer (A) and monomer (B) obtained using Superose 6. Elution volume and molar mass (MM) for D8 Δ262 oligomer (C) and monomer (D) obtained using Superdex S200. The horizontal dark line under each peak corresponds to the MM of the eluting sample as determined by SEC-UV/MALS. E. Reported SEC-UV/MALS MMs. (TIF) [file ppat.1004495.s003.tif]

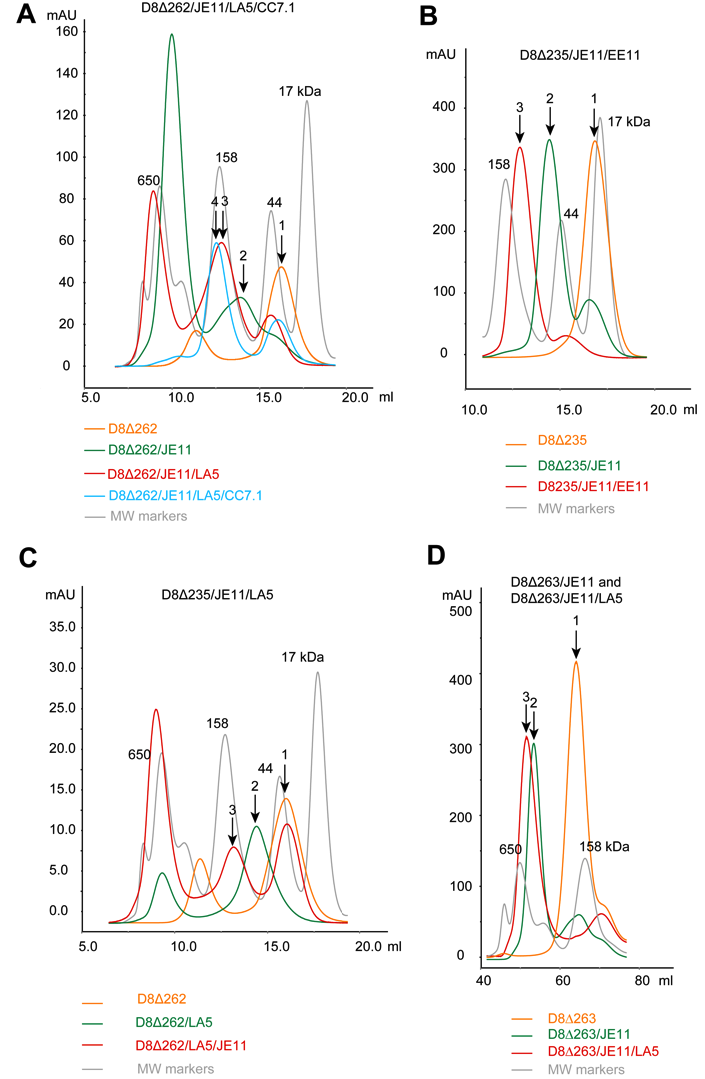

Supplement: Figure S8 — Preparation of monomeric and oligomeric D8/Fab complexes. A, B, and C. D8-monomer complexes. Associated class averages can be seen in figures S5, S6, and S7 D. D8-hexamer complexes. Associated class averages of unliganded and Fab-bound D8 hexamers can be seen in figure S4. All complexes are prepared by performing recursive SEC runs, starting by purifying the D8 monomer or D8 hexamer, and performing subsequent SEC runs for each additional Fab added to the complex being prepared. Curves are colored according to their order in the sequential process (SEC #1: orange, SEC#2: green, SEC #3: red, SEC#4: cyan). MWapp markers in kDa are shown for reference (grey curve). Based on the cross-blocking data, we also built the quaternary complex D8/JE11/CC4.1/LA5 (panel a, SEC#4). Existence of this complex is evidenced by the class average in figure S9. (TIF) [file ppat.1004495.s008.tif]
